# Cytoglobin as a Biomarker in Cancer: Potential Perspective for Diagnosis and Management

**DOI:** 10.1155/2015/824514

**Published:** 2015-08-03

**Authors:** Tatsha C. Bholah, Vidushi S. Neergheen-Bhujun, Nikolas J. Hodges, Sabrina D. Dyall, Theeshan Bahorun

**Affiliations:** ^1^Department of Biosciences, Faculty of Science, University of Mauritius, 80837 Réduit, Mauritius; ^2^ANDI Centre of Excellence for Biomedical and Biomaterials Research, University of Mauritius, 80837 Réduit, Mauritius; ^3^Department of Health Sciences, Faculty of Science, University of Mauritius, 80837 Réduit, Mauritius; ^4^School of Biosciences, University of Birmingham, Edgbaston, Birmingham B15 2TT, UK

## Abstract

The search for biomarkers to detect the earliest glimpse of cancer has been one of the primary objectives of cancer research initiatives. These endeavours, in spite of constant clinical challenges, are now more focused as early cancer detection provides increased opportunities for different interventions and therapies, with higher potential for improving patient survival and quality of life. With the progress of the omics technologies, proteomics and metabolomics are currently being used for identification of biomarkers. In this line, cytoglobin (Cygb), a ubiquitously found protein, has been actively reviewed for its functional role. Cytoglobin is dynamically responsive to a number of insults, namely, fibrosis, oxidative stress, and hypoxia. Recently, it has been reported that Cygb is downregulated in a number of malignancies and that an induced overexpression reduces the proliferative characteristics of cancer cells. Thus, the upregulation of cytoglobin can be indicative of a tumour suppressor ability. Nevertheless, without a comprehensive outlook of the molecular and functional role of the globin, it will be most unlikely to consider cytoglobin as a biomarker for early detection of cancer or as a therapeutic option. This review provides an overview of the proposed role of cytoglobin and explores its potential functional role as a biomarker for cancer and other diseases.

## 1. Introduction

**Figure 1:**
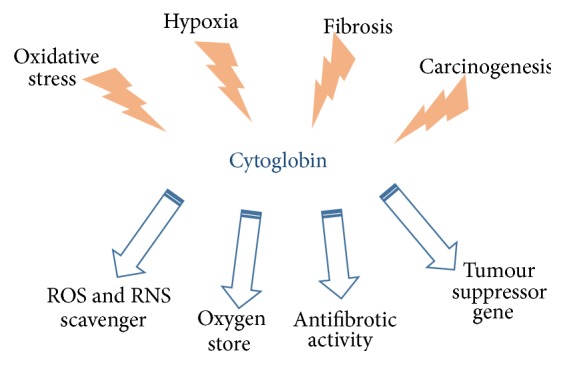
Potential functional roles of cytoglobin in response to insults. Such activities inspire further investigation for clinical applications.

Cytoglobin (Cygb) was discovered more than a decade ago in a proteomic screen of fibrotic liver by a group of researchers in Japan and was originally named STAP (Stellate Activating Protein) [1]. Since its discovery, many studies have been conducted to comprehend its functional role but the latter still remains presently poorly understood. Due to phylogenetic and structural similarities with other globins (myoglobin, haemoglobin, and neuroglobin), it was rapidly classified as a member of the globin family. This classification led researchers to suggest a putative role for Cygb as a respiratory protein similar to the other well characterised globins known to exist at the time, that is, haemoglobin (Hb) and myoglobin (Mb). Ubiquitously present in cells, Cygb appears to play a more universal role than that of Hb, Mb, and neuroglobin (Ngb, another recently identified globin), which are specifically found in red blood cells, muscle cells, and cells of the central nervous system, respectively. Interestingly, Cygb has been shown to exhibit many respiratory roles in normal cells including oxygen storage, reactive oxygen species (ROS) scavenging, terminal oxidase activity, and antifibrotic activities [2–7]. Its role in respiration has been reviewed, owing to its relationship with the globin family and also due to its upregulation in hypoxia [4, 8–10], with however no specific outcome to determine its exact role. More recently, Cygb has been reported to have some implications in cancer. In most cancer cells, Cygb expression is downregulated by hypermethylation, showing an epigenetic control [11, 12]. This downregulation in cancer cells prompts suggesting a possible role as a tumour suppressor gene (TSG) [13]. Conversely, in a few malignancies, Cygb is upregulated [14] where this stimulus is likely to be related to resistance to hypoxia. In line with on-going research in this field, this commentary paper is being proposed to debate the putative role of Cygb and to provide a perspective on potential research areas that may point out its role as a cancer biomarker (Figure 1).

## 2. Respiratory Functions of Cygb

Cygb is a globin protein, expressed in various tissues including liver, heart, stomach, lungs, spleen, and muscles, where its physiological function remains to be defined [1, 8, 13]. It is a 190-amino acid hexacoordinated hemeprotein of the globin family with a molecular weight of 20.9 kDa [10, 15]. Being quite similar to Mb in orientation, span, and primary structure, it has a distinct intramolecular disulphide bond and heme-coordination [4, 16–18]. Molecular phylogenetic studies point towards an ancient origin and highly conserved biological function for cytoglobin, supported by its slower amino acid mutation rate, compared to the other globins [19]. In the light of current available reports and also due to its part similarity to other globins (Mb and Ngb), several possible cellular functions of Cygb have been considered in line with respiratory activities. These include oxygen storage, terminal oxidase activity, and reactive oxygen species (ROS) scavenging [4, 20, 21]. Though the enzymatic activity of cytoglobin has been suggested, this remains controversial. There is limited evidence of catalase, peroxidase, and superoxide dismutase (SOD) activity due to very low reported quantitative levels [22].

The oxygen carrier and storage function of Cygb was suggested based on structural similarities with myoglobin and stimulation of its expression under hypoxic conditions [8, 15, 18]. It is hypothesized that Cygb acts as an oxygen source because of its high affinity for oxygen (about 1 Torr) and also due to its pH-dependent oxygen binding ability similar to Mb, though unlike Ngb [3, 23–25]. The cooperative binding of oxygen to Cygb-heme supports the ease of its loading and unloading over a narrow range of oxygen pressures/tension compared to noncooperative binding as in Ngb and Mb. However, the heme-heme interaction for cooperative binding remains still to be described to illustrate clearly its function in respiration [19, 24]. Nevertheless, the fact that Cygb is hexacoordinated unlike Mb and Hb strongly argues against a simple oxygen binding function. In the absence of exogenous ligands, Cygb takes up its original structure by binding Fe to the endogenous ligand, HisE7. Upon reduction of the hemeprotein, the affinity for exogenous ligands such as O_2_, NO, and CO decreases. This makes binding of a forthcoming ligand even more difficult [16]. Thus the oxygen storage ability of Cygb is debatable. Additionally, its cellular localisation and level of cellular expression do not support specific reaction within mitochondria in a way analogous to Mb [26]. Consequently, it can be perceived that cytoglobin may probably act in a different manner to sustain respiratory activities in cells.

Indeed, it has been shown that Cygb's overexpression in anoxic and hypoxic conditions sustains survival of cells and its low expression in similar conditions led to increasing apoptosis of control cells [27–30]. Its gene contains a long CpG island upstream, with several transcription sites including hypoxia responsive elements (HRE) and hypoxia-inducible protein binding sites among others (HIF1, AP1, AP2, and SP1), thereby providing the basis of cytoglobin regulation in hypoxia [13, 31]. Regulation by HIF1*α* (hypoxia-inducible factor 1 *α*) shows significant increase in its expression [28, 32]. In the same line, it has been suggested that Cygb donates oxygen for hydroxylation of HIF1*α* to enhance its destabilisation in ischemic conditions. Thus the cell could bypass the sensing of hypoxia and apoptosis, ultimately leading to cell survival [19].

Furthermore, antiapoptotic pathway in ischemic conditions also involves cytochrome c (cyt c). In a study by Fago et al., Ngb was shown to be able to reduce the iron centre of cyt c preventing association with and activation of caspase 9 and could therefore be considered an “antiapoptotic” factor. Although at the moment there is no evidence that Cygb could function in an analogous manner this cannot be excluded and warrants further investigation. This hypothesis is further supported by similarities between the two proteins, being both hexacoordinated and having similar oxygen affinities [20]. A potential reaction of Cygb with mitochondria cyt c, as reported for Ngb [20], could also account for a respiratory or antiapoptotic activity. This further illustrates its respiratory functions in hypoxic conditions, without being directly involved with oxygen chemistry.

## 3. Cygb in Oxidative Stress

Together with upregulation of Cygb in hypoxia, Cygb is also overexpressed under conditions of oxidative stress (OS) [32, 33]. A significant increase in Cygb occurred in neuroblastoma cells when the latter were subjected to hydrogen peroxide (H_2_O_2_) [32]. During similar ROS insults, fibrotic cells such as hepatic stellate cells (HSCs) also upregulated their Cygb expression to neutralize the free radicals [34, 35]. In the same line, cytoglobin has hexacoordinated structure, similar to Ngb, and cyt c hemeproteins, which provide redox reaction abilities through their hemes [20]. These reports emphasize the protective role of Cygb, more particularly its radical scavenging function, as evidenced by its ability to detoxify radicals via reactions with its heme group [36]. Cygb also shows lipid peroxidase activity through its heme group. Since products of lipid peroxidation are known to modulate cell signalling, the low levels of Cygb in cells might be liable to induce protective actions by means of the peroxidase activities in oxidative stress conditions [37]. However, the role of cytoglobin in OS is still to be discovered as the mechanisms of upregulation and protective role are still debatable. Recently, Verschoor and Singh have shown that an overexpression of Ets-1 has decreased ROS insults in cells. While an Ets-1 binding site is present at the promoter region of Cygb, no link was pursued in line with upregulation of Cygb [31, 38]. It can be anticipated that increase in Ets-1 has upregulated Cygb level in the cells which has consequently decreased the ROS levels, providing an inspiring target of research towards the signalling pathway for Cygb expression.

**Figure 2:**
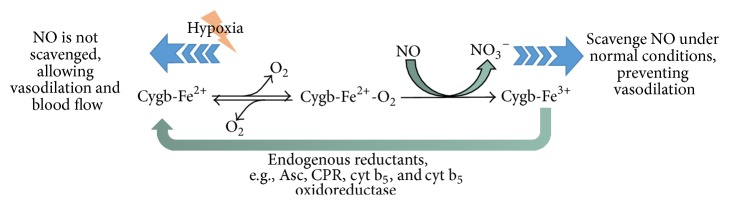
Illustrative diagram showing the regulation of NO level by cytoglobin.

Cygb has been shown to be protective not only against ROS but also against RNS (Reactive Nitrogen Species) such as nitric oxide (NO), an endogenously present molecule known to cause cellular and DNA damage [2, 39]. The rate of metabolism of NO is O_2_-dependent in both vascular and intervascular tissues, where Mb is deficient [40, 41]. Interestingly, Cygb has been reported to have a NO dioxygenase (NOD) activity similar to Mb and Ngb [2]. Though the level of Cygb in these cells is low (micromolar) [17, 19], it is noteworthy to reflect upon how Cygb could act as a NOD at this low level in dramatic inductions of NO in cells. This could be plausibly explained by considering that Cygb does not actually scavenge NO but regulates its levels in hypoxic conditions. Liu and colleagues [40] have reported that the rate of NO metabolism increases significantly in high O_2_ level and decreases in low O_2_ concentration. NO is a known endogenous molecule which has an important regulatory role in the dilation of blood vessels [42]. Therefore, Cygb can be suggested to indirectly regulate vascular tone in tissues under hypoxia by increasing the diameter of blood vessels, thereby alleviating the oxygen deficiency stress (Figure 2). This proposed mechanism can be depicted as follows: Cygb binds to oxygen at high PO_2_ and donates its oxygen to scavenge NO to NO_3_^−^, forming met-Cygb (Fe^3+^) [40]. In turn, the met-Cygb may be reduced by many endogenous reducing agents such as ascorbic acid, cytochrome P450 reductase (CPR), cytochrome b_5_, and cytochrome b_5_ oxidoreductase [40, 43]. The reduction of Cygb enables the recycling of the hemeprotein, powering an efficient regulation of NO even at a low expression level. Congruently, further analyses reveal that cellular reductants greatly increase the rate of NO metabolism in the presence of oxygen [40]. Following these reports, Cygb seems therefore to be an eligible candidate in regulating vascular tone in hypoxia. The underlying molecular mechanism of O_2_-dependent NO regulation by Cygb and its intermediates (cygb-Fe^3+^) remains however still uncertain and provides a potential perspective for future research.

## 4. Role in Carcinogenesis

Advances in the domain of molecular research have attributed another interesting function to Cygb: its ability to suppress tumour growth. Changes in Cygb expression have been shown to occur in many malignancies. Investigations on Cygb's tumour suppressing activity reported in 2006 have shown that most cancer cells have a reduced expression of Cygb, with a dramatic decrease (70%) of cytoglobin expression reported in tylosis with oesophageal cancer (TOC) [12, 44, 45]. Furthermore, studies have reported reduction in tumor growth with an overexpression of Cygb by the transfection of cytoglobin cDNA in non-small lung cancer cells and breast cancer cells [12]. In another study, knockdown of Cygb in glioma cells showed an increase in growth rate of the cells [46]. These reports strongly suggest a tumour suppressor activity of Cygb. Findings also show that NO and peroxynitrite (ONOO^−^) produced during inflammatory responses can affect tumour suppressor proteins such as p53, mitogen-activated protein kinase pathways in dose-dependent manner to promote damage to DNA, and genetic library and cancer phenotypes of cells [39, 47]. Since in inflammation there is a global hypermethylation mostly in tumor suppressor genes [48], Cygb expression might also be affected in a similar manner. Furthermore, it has also been reported that Cygb's protective function against ROS was also extended to prevent cell death and ROS induced genetic damage in cells [49].

In the same line, loss of expression of cytoglobin in cancer cells occurs due to loss of heterozygosity (LOH) and also by epigenetic regulation via hypermethylation of CpG islands in the Cygb gene promoter region [44, 45, 50, 51]. In addition, differences in hypermethylation levels were observed in lung adenocarcinoma and lung squamous cell carcinoma [50] with however an unclear relationship between the quantitative level of hypermethylation and stages of carcinogenesis [12]. The identification of a marker that assesses the possibility for cells to become cancerous and for cancerous cells to further develop remains of vital importance in cancer research. This link between the quantitative determinations of hypermethylation levels of Cygb promoter gene in different cancer cells could draw attention to the role of Cygb as a biomarker for carcinogenesis notably in determining the cancer level and the likelihood of cells to further developing phenotypes of cancer.

Also, studies conducted on Cygb-deficient mice have been shown to bridge the link between the role of the ROS scavenging and tumour repressing activities of Cygb, whereby susceptibility to tumorigenesis was increased in Cygb-deficient mice on treatment with DEN (*N*,*N*-diethylnitrosamine**)** [52]. Altogether, Cygb might exhibit both TSG and oncogene features. Contradicting most results, a small subset of malignant samples showed a high mRNA expression of Cygb [14], which is thought to arise from a different mechanistic pathway. Further investigations in this arena will certainly bring to light the eventual functional role of Cygb in cancer. In the same vein, the promising characteristics of Cygb may provide the basis for a prospective role in clinical treatments.

## 5. Role in Fibrosis

Cytoglobin, first named STAP (stellate cell activation-associated protein), was initially located in HSCs (hepatic stellate cells) during their activation upon liver fibrosis. The level of expression of Cygb was therein found to be elevated. In a functional point of view, expression of Cygb was suggested to support HSC in liver traumas where they are increasingly exposed to endogenous ROS. In that way, it was presumed to act as a potential ROS scavenger counteracting the increasing ROS insults [34]. Further evidences have led to suggesting a potential role of Cygb as an oxygen donor in the synthesis of collagen. Schmidt and colleagues have previously speculated the role of Cygb as an oxygen donor for the enzyme prolyl-4-hydroxylase in collagen synthesis, suggesting role of Cygb as a profibrotic globin [15]. In further support, Man et al. published reports with a view to elucidating the role of Cygb in collagen metabolism. In the study, liver tissues were subjected to hepatotoxin, CCl_4_. 24 hrs after the exposure, Cygb was upregulated, followed by the upregulation of procollagen-*α*-1 expression 48 hrs later [35]. From these data, it is most probable that Cygb upregulation is associated with fibrosis, but the role of Cygb as profibrotic or antifibrotic globin remains still ambiguous.

In contrast to the above, accruing evidences point towards antifibrotic roles for Cygb. It was observed that Cygb upregulation in an induced fibrotic damaged kidney led to an improvement in the physiology of the organ [6]. Similar results were reported where transfection of Cygb gene in a CCl_4_-induced fibrotic rat liver had resulted in physiologic remodelling and decreased fibrosis of the liver tissue. Cygb overexpression in these cells inhibited the anticipated upregulation of several fibrosis-associated components such as, procollagen-1, TGF-*β*1, TIMP-1 transcripts and *α*-SMA and TGF-*β*1 proteins [7]. Although the specific mechanism of inhibition was not elucidated, it was reported that antifibrotic activity originated from the heme group within Cygb [6]. There is evidently an association between Cygb and fibrosis, but the mechanism is largely unexplored. Elucidation of this association may potentially lead to development of Cygb-based therapies for fibrosis.

## 6. Future Perspectives

So finally, after more than a decade from the discovery of cytoglobin, where are we? Many studies in diverse arenas have been conducted to clarify the role of cytoglobin. Many putative functions have also been proposed. Along most of these suggested functions are contradicting comment and reports. Neither the oxygen storage ability nor the ROS/RNS scavenging function and not even the tumour suppressor activity of cytoglobin has been strongly accepted. This challenge is still on, as Cygb instigates increasing interest and curiosity as evidenced by very recent publications. As such, Chakraborty et al. are proposing a potential link between the proposed activities of Cygb: ROS scavenger, antifibrotic and anticancer abilities [53]. In the quest of unanswered questions, the antifibrotic activity of Cygb needs to be further exploited in line with preliminary data advocating its anticancer therapy. While, in tumor development and propagation, several biological and cellular changes occur, differential levels of hypermethylation of the CpG island of the Cygb promoter region have been reported. Detailed understanding of the level of hypermethylation at different stages of tumor development is of clinical significance to identify Cygb as a novel biomarker of cancer. Another point that requires elucidation is whether Cygb has a tumor suppressor or an oncogenic ability. Further molecular research is of notable importance to identify trigger switches responsible for the transition of Cygb's function in different biological insults (fibrosis, hypoxia, oxidative stress, and cancer). Recently, Cui et al. have reported a first compound, arundic acid, identified to upregulate Cygb. Similar investigations are needed to expand the molecular pathway of modulating the endogenous levels of Cygb [54]. These future investigations are strongly warranted with a view to strongly identifying Cygb as a potential biomarker, prognosis, and potential therapy for medical use.

## References

[1] Asahina K., Kawada N., Kristensen D. B. (2002). Characterization of human stellate cell activation-associated protein and its expression in human liver. *Biochimica et Biophysica Acta—Gene Structure and Expression*.

[2] Halligan K. E., Jourd'heuil F. L., Jourd'heuil D. (2009). Cytoglobin is expressed in the vasculature and regulates cell respiration and proliferation via nitric oxide dioxygenation. *The Journal of Biological Chemistry*.

[3] Hamdane D., Kiger L., Dewilde S. (2003). The redox state of the cell regulates the ligand binding affinity of human neuroglobin and cytoglobin. *The Journal of Biological Chemistry*.

[4] Hankeln T., Ebner B., Fuchs C. (2005). Neuroglobin and cytoglobin in search of their role in the vertebrate globin family. *Journal of Inorganic Biochemistry*.

[5] He X., Lv R., Wang K. (2011). Cytoglobin exhibits anti-fibrosis activity on liver in vivo and in vitro. *The Protein Journal*.

[6] Mimura I., Nangaku M., Nishi H., Inagi R., Tanaka T., Fujita T. (2010). Cytoglobin, a novel globin, plays an antifibrotic role in the kidney. *American Journal of Physiology—Renal Physiology*.

[7] Xu R., Harrison P. M., Chen M. (2006). Cytoglobin overexpression protects against damage-induced fibrosis. *Molecular Therapy*.

[8] Burmester T., Ebner B., Weich B., Hankeln T. (2002). Cytoglobin: a novel globin type ubiquitously expressed in vertebrate tissues. *Molecular Biology and Evolution*.

[9] Burmester T., Gerlach F., Hankeln T. (2007). Regulation and role of neuroglobin and cytoglobin under hypoxia. *Hypoxia and the Circulation*.

[10] Pesce A., Bolognesi M., Bocedi A. (2002). Neuroglobin and cytoglobin. Fresh blood for the vertebrate globin family. *EMBO Reports*.

[11] McRonald F. E., Risk J. M., Hodges N. J. (2012). Protection from intracellular oxidative stress by cytoglobin in normal and cancerous oesophageal cells. *PLoS ONE*.

[12] Shivapurkar N., Stastny V., Okumura N. (2008). Cytoglobin, the newest member of the globin family, functions as a tumor suppressor gene. *Cancer Research*.

[13] Oleksiewicz U., Liloglou T., Field J. K., Xinarianos G. (2011). Cytoglobin: biochemical, functional and clinical perspective of the newest member of the globin family. *Cellular and Molecular Life Sciences*.

[14] Chen H., Zhao X., Meng T. (2014). Expression and biological role of cytoglobin in human ovarian cancer. *Tumor Biology*.

[15] Schmidt M., Gerlach F., Avivi A. (2004). Cytoglobin is a respiratory protein in connective tissue and neurons, which is up-regulated by hypoxia. *Journal of Biological Chemistry*.

[16] de Sanctis D., Dewilde S., Pesce A. (2004). Crystal structure of cytoglobin: the fourth globin type discovered in man displays heme hexa-coordination. *Journal of Molecular Biology*.

[17] Lechauve C., Chauvierre C., Dewilde S. (2010). Cytoglobin conformations and disulfide bond formation. *The FEBS Journal*.

[18] Sugimoto H., Makino M., Sawai H., Kawada N., Yoshizato K., Shiro Y. (2004). Structural basis of human cytoglobin for ligand binding. *Journal of Molecular Biology*.

[19] Fago A., Hundahl C., Malte H., Weber R. E. (2004). Functional properties of neuroglobin and cytoglobin. Insights into the ancestral physiological roles of globins. *IUBMB Life*.

[20] Fago A., Mathews A. J., Moens L., Dewilde S., Brittain T. (2006). The reaction of neuroglobin with potential redox protein partners cytochrome b5 and cytochrome c. *FEBS Letters*.

[21] Kiger L., Tilleman L., Geuens E. (2011). Electron transfer function versus oxygen delivery: a comparative study for several hexacoordinated globins across the animal kingdom. *PLoS ONE*.

[22] Trandafir F., Hoogewijs D., Altieri F. (2007). Neuroglobin and cytoglobin as potential enzyme or substrate. *Gene*.

[23] Emara M., Turner A. R., Allalunis-Turner J. (2010). Hypoxic regulation of cytoglobin and neuroglobin expression in human normal and tumor tissues. *Cancer Cell International*.

[24] Fago A., Hundahl C., Dewilde S., Gilany K., Moens L., Weber R. E. (2004). Allosteric regulation and temperature dependence of oxygen binding in human neuroglobin and cytoglobin: molecular mechanisms and physiological significance. *The Journal of Biological Chemistry*.

[25] Trent J. T., Hargrove M. S. (2002). A ubiquitously expressed human hexacoordinate hemoglobin. *The Journal of Biological Chemistry*.

[26] Nakatani K., Okuyama H., Shimahara Y. (2004). Cytoglobin/STAP, its unique localization in splanchnic fibroblast-like cells and function in organ fibrogenesis. *Laboratory Investigation*.

[27] Fordel E., Geuens E., Dewilde S., De Coen W., Moens L. (2004). Hypoxia/ischemia and the regulation of neuroglobin and cytoglobin expression. *IUBMB Life*.

[28] Fordel E., Geuens E., Dewilde S. (2004). Cytoglobin expression is upregulated in all tissues upon hypoxia: an in vitro and in vivo study by quantitative real-time PCR. *Biochemical and Biophysical Research Communications*.

[29] Fordel E., Thijs L., Martinet W., Schrijvers D., Moens L., Dewilde S. (2007). Anoxia or oxygen and glucose deprivation in SH-SY5Y cells: a step closer to the unraveling of neuroglobin and cytoglobin functions. *Gene*.

[30] Singh S., Canseco D. C., Manda S. M. (2014). Cytoglobin modulates myogenic progenitor cell viability and muscle regeneration. *Proceedings of the National Academy of Sciences of the United States of America*.

[31] Guo X., Philipsen S., Tan-Un K.-C. (2006). Characterization of human cytoglobin gene promoter region. *Biochimica et Biophysica Acta—Gene Structure and Expression*.

[32] Li D., Chen X. Q., Li W.-J., Yang Y.-H., Wang J.-Z., Yu A. C. H. (2007). Cytoglobin up-regulated by hydrogen peroxide plays a protective role in oxidative stress. *Neurochemical Research*.

[33] Mammen P. P. A., Shelton J. M., Ye Q. (2006). Cytoglobin is a stress-responsive hemoprotein expressed in the developing and adult brain. *Journal of Histochemistry & Cytochemistry*.

[34] Kawada N., Kristensen D. B., Asahina K. (2001). Characterization of a stellate cell activation-associated protein (STAP) with peroxidase activity found in rat hepatic stellate cells. *The Journal of Biological Chemistry*.

[35] Man K.-N. M., Philipsen S., Tan-Un K. C. (2008). Localization and expression pattern of cytoglobin in carbon tetrachloride-induced liver fibrosis. *Toxicology Letters*.

[36] Nishi H., Inagi R., Kawada N. (2011). Cytoglobin, a novel member of the globin family, protects kidney fibroblasts against oxidative stress under ischemic conditions. *The American Journal of Pathology*.

[37] Reeder B. J., Svistunenko D. A., Wilson M. T. (2011). Lipid binding to cytoglobin leads to a change in haem co-ordination: a role for cytoglobin in lipid signalling of oxidative stress. *The Biochemical Journal*.

[38] Verschoor M. L., Verschoor C. P., Singh G. (2013). Ets-1 global gene expression profile reveals associations with metabolism and oxidative stress in ovarian and breast cancers. *Cancer & Metabolism*.

[39] Ying L., Hofseth L. J. (2007). An emerging role for endothelial nitric oxide synthase in chronic inflammation and cancer. *Cancer Research*.

[40] Liu X., Follmer D., Zweier J. R. (2012). Characterization of the function of cytoglobin as an oxygen-dependent regulator of nitric oxide concentration. *Biochemistry*.

[41] Liu X., Tong J., Zweier J. R. (2013). Differences in oxygen-dependent nitric oxide metabolism by cytoglobin and myoglobin account for their differing functional roles. *The FEBS Journal*.

[42] Förstermann U., Münzel T. (2006). Endothelial nitric oxide synthase in vascular disease: from marvel to menace. *Circulation*.

[43] Gardner A. M., Cook M. R., Gardner P. R. (2010). Nitric-oxide dioxygenase function of human cytoglobin with cellular reductants and in rat hepatocytes. *The Journal of Biological Chemistry*.

[44] McRonald F. E., Liloglou T., Xinarianos G. (2006). Down-regulation of the cytoglobin gene, located on 17q25, in tylosis with oesophageal cancer (TOC): evidence for trans-allele repression. *Human Molecular Genetics*.

[45] Moodley R., Reddi A., Chetty R., Naidoo R. (2007). Abnormalities of chromosome 17 in oesophageal cancer. *Journal of Clinical Pathology*.

[46] Fang J., Ma I., Allalunis-Turner J. (2011). Knockdown of cytoglobin expression sensitizes human glioma cells to radiation and oxidative stress. *Radiation Research*.

[47] Pacher P., Beckman J. S., Liaudet L. (2007). Nitric oxide and peroxynitrite in health and disease. *Physiological Reviews*.

[48] Kundu J. K., Surh Y.-J. (2008). Inflammation: gearing the journey to cancer. *Mutation Research—Reviews in Mutation Research*.

[49] Hodges N. J., Innocent N., Dhanda S., Graham M. (2008). Cellular protection from oxidative DNA damage by over-expression of the novel globin cytoglobin in vitro. *Mutagenesis*.

[50] Xinarianos G., McRonald F. E., Risk J. M. (2006). Frequent genetic and epigenetic abnormalities contribute to the deregulation of cytoglobin in non-small cell lung cancer. *Human Molecular Genetics*.

[51] Shaw R. J., Omar M. M., Rokadiya S. (2009). Cytoglobin is upregulated by tumour hypoxia and silenced by promoter hypermethylation in head and neck cancer. *British Journal of Cancer*.

[52] Thuy L. T. T., Morita T., Yoshida K. (2011). Promotion of liver and lung tumorigenesis in DEN-treated cytoglobin-deficient mice. *The American Journal of Pathology*.

[53] Chakraborty S., John R., Nag A. (2014). Cytoglobin in tumor hypoxia: novel insights into cancer suppression. *Tumor Biology*.

[54] Cui W., Wang M., Maegawa H., Teranishi Y., Kawada N. (2012). Inhibition of the activation of hepatic stellate cells by arundic acid via the induction of cytoglobin. *Biochemical and Biophysical Research Communications*.

